# *SPAG9* Expression Predicts Good Prognosis in Patients with Clear-Cell Renal Cell Carcinoma: A Bioinformatics Analysis with Experimental Validation

**DOI:** 10.3390/genes14040944

**Published:** 2023-04-20

**Authors:** Liwen Qiao, Lu Zhang, Huiming Wang

**Affiliations:** Department of Nephrology, Renmin Hospital of Wuhan University, Wuhan 430060, China; qiaoliwen2021@163.com (L.Q.);

**Keywords:** *SPAG9*, autophagy, inflammation, clear cell renal carcinoma, nomograms

## Abstract

Clear-cell renal cell carcinoma (ccRCC) is the most common and aggressive type of renal-cell carcinoma (RCC). Sperm-associated antigen 9 (*SPAG9*) has been reported to promote the progression of a variety of tumors and is thus a potential prognostic marker. This study combined a bioinformatics analysis with an experimental validation, exploring the prognostic value of *SPAG9* expression in ccRCC patients and the possible underlying mechanisms. The *SPAG9* expression was associated with a poor prognosis in pan-cancer patients, but with a good prognosis and slow tumor progression in ccRCC patients. To explore the underlying mechanism, we investigated the roles of *SPAG9* in ccRCC and bladder urothelial carcinoma (BLCA). The latter was chosen for comparison with ccRCC to represent the tumor types in which *SPAG9* expression suggests a poor prognosis. The overexpression of *SPAG9* increased the expression of autophagy-related genes in 786-O cells but not in HTB-9 cells, and *SPAG9* expression was significantly correlated with a weaker inflammatory response in ccRCC but not in BLCA. Through an integrated bioinformatics analysis, we screened out seven key genes (*AKT3, MAPK8, PIK3CA, PIK3R3, SOS1, SOS2,* and *STAT5B*) in this study. The correlation between *SPAG9* expression and ccRCC prognosis depends on the expression of key genes. Since most of the key genes were PI3K-AKT-pathway members, we used the PI3K agonist 740Y-P to stimulate the 786-O cells, to mimic the effect of key-gene overexpression. Compared with the Ov-*SPAG9* 786-O cells, the 740Y-P further increased the expression of autophagy-related genes by more than twofold. Moreover, we constructed a nomogram based on *SPAG9*/key genes and other clinical features, which was proven to have some predictive value. Our study found that *SPAG9* expression predicted opposite clinical outcomes in pan-cancer and ccRCC patients, and we speculated that *SPAG9* suppresses tumor progression by promoting autophagy and inhibiting inflammatory responses in ccRCC. We further found that some genes might cooperate with *SPAG9* to promote autophagy, and that these were highly expressed in the tumor stroma and could be represented by key genes. The *SPAG9*-based nomogram can help to estimate the long-term prognosis of ccRCC patients, indicating that *SPAG9* is a potential prognostic marker for ccRCC.

## 1. Introduction

Renal cell carcinoma (RCC) is the most common adult renal malignancy, of which the most common and aggressive histologic type is clear-cell renal cell carcinoma (ccRCC). Clear-cell renal cell carcinoma is characterized by abundant glycogen and lipids in the cytoplasm, accounting for 80–90% of RCC cases [1]. The incidence of ccRCC has increased steadily in recent decades, and due to its lack of specific clinical manifestations, nearly one-third of ccRCC patients have already developed distant metastasis at the time of diagnosis and, thus, missed the best opportunity for surgery [2]. Clear-cell renal cell carcinoma is insensitive to chemotherapy or radiotherapy, and although molecular targeted therapies have been widely used in patients with advanced ccRCC, most patients develop drug resistance after 5–11 months [3]. Therefore, it is necessary to investigate the molecular mechanisms underlying the occurrence and development of ccRCC and to develop potential therapeutic targets and prognostic markers. 

Sperm-associated antigen 9 (*SPAG9*) is a cancer-testis antigen expressed in various tumor tissues, which has two main alternative spliceosomes—JLP (1307 amino acids) and SPAG9 (766 amino acids). It plays a wide role in tumor-cell lines: it not only promotes proliferation, migration, and epithelial–mesenchymal transition (EMT) [4,5,6,7,8,9,10,11,12,13,14,15,16,17], but also regulates autophagy [18,19]. A previous study found that *SPAG9* overexpression promotes proliferation and migration in the ccRCC cell lines Caki-1 and NII-AKS413 [4]; however, studies on the prognostic value of *SPAG9* in ccRCC patients are still lacking. 

This study combined a bioinformatics analysis and experimental validation, exploring the prognostic value of *SPAG9* expression in ccRCC patients and the possible underlying mechanisms, and constructed a *SPAG9*-based ccRCC prognostic model to help with risk stratification.

## 2. Materials and Methods

### 2.1. Data Acquisition

The RNA-seq data and clinical data of ccRCC patients were downloaded from the TCGA database (https://portal.gdc.cancer.gov/ accessed on 15 June 2022). Gene expression was shown as FPKM (fragments per kilobase of transcript per million mapped fragments). The clinical data included age, gender, pathological grade (G1~G4), clinical stage (Stage I~Stage IV), T stage (tumor size, T1~T4), N stage (tumor lymph-node metastasis, N0~N1), and M stage (tumor distant metastasis, M0~M1). The clinical data of ccRCC included 537 patients, 526 of whom had complete and valid survival information (survival time and vital status), for Kaplan–Meier survival analysis, and 515 of whom had complete and valid survival information and clinical information (age, gender, pathological grade and clinical stage), for Cox regression analysis. 

We also obtained RNA-seq data and clinical data of ccRCC from the ICGC database (https://dcc.icgc.org/ accessed on 15 June 2022): RECA-EU, including 91 patients with complete and valid survival information (survival time and vital status) and clinical information (age, gender). Basic information of the ccRCC patients from the TCGA database and the ICGC database is shown in Tables S1 and S2. 

The scRNA-seq data were obtained from the GEO database (https://www.ncbi.nlm.nih.gov/geo/ accessed on 1 June 2022). Two ccRCC samples (GSM4630028 and GSM4630028) and one normal kidney-tissue sample (GSM4630031) were derived from the GSE152938 dataset, with a reading depth of 10× Genomics based on HiSeq X Ten (Illumina, San Diego, CA, USA) [20]. All 3 scRNA-seq samples were from human organisms.

### 2.2. Analysis with the Online Tool

The GEPIA2 is an online analysis tool developed by Tang et al., based on the data from the TCGA database (http://gepia2.cancer-pku.cn/ accessed on 15 June 2022) [21]. Some of the survival curves were established by GEPIA2: after entering gene names, median gene expression was selected as the group cutoff, and the overall survival (OS) rate of the 2 groups was compared by log-rank test. The online tool calculated the hazard ratios (HRs) based on the Cox PH Model, and 95% confidence interval (95% CI) was shown as dotted lines in the pictures. Axis units were set to months. Some correlation analyses were also performed by GEPIA2: after entering gene names, and Pearson’s correlation coefficient between the expressions of genes was calculated.

### 2.3. Cell Culture and RNA Transfection

The human ccRCC cell line 786-O and BLCA cell line HTB-9 were provided by the American Type Culture Collection (ATCC, Manassas, VA, USA). Cells were cultured in DMEM-F12 medium containing 10% fetal bovine serum (FBS) and kept at 37 °C in a cell incubator with 5% CO_2_ and 95% air. Overexpression of *SPAG9* (Ov-*SPAG9*) and overexpression control (Ov-NC) were synthesized by RIBIO (Guangzhou, China). Transfection was carried out using Lipofectamine 3000 (Invitrogen, Waltham, MA, USA), following the manufacturer’s protocol. After 48 h, the transfection efficiency was detected by real-time quantitation PCR (RT–qPCR). In addition, 786-O cells were treated with the PI3K agonist 740Y-P (20 µM; MedChemExpress, Monmouth Junction, NJ, USA) for 48 h.

### 2.4. RNA Extraction and RT–qPCR Assay

Total RNA from cells was extracted using TRIzol reagent (Invitrogen) and reverse-transcribed into cDNA using a RevertAid cDNA Synthesis Kit (Thermo Fisher Scientific, Waltham, MA, USA). The RT–qPCR was performed with SYBR Green PCR Master Mix (Illumina), according to the manufacturer’s instructions. Relative mRNA levels were measured with the aid of the 2^−ΔΔCt^ method and standardized to GAPDH. The primer sequences and the qPCR data are shown in Tables S3 and S4.

### 2.5. GSEA and ssGSEA Analysis

Gene set enrichment analysis (ssGSEA) algorithm was used to evaluate the inflammatory response, inflammatory factor production, and T-cell-exhaustion levels in ccRCC and BLCA samples. The gene set “GOBP_INFLAMMATORY_RESPONSE” from the Molecular Signatures database (http://www.gsea-msigdb.org/gsea/msigdb/index.jsp/ accessed on 15 June 2022) was used to score the inflammatory response. Inflammatory factors that promote tumor progression (*CRP*, *IL1A*, *IL1B*, *IL6*, *TNF*, *TGFB*, *VEGFA*, *VEGFB*, *VEGFC*, *CXCL1*, *CXCL2*, *MMP2*, and *MMP9*) were used to score inflammatory factor production [22]; T-cell-exhaustion markers (*PDCD1*, *TOX*, *CXCL13*, *TIGIT*, *CTLA4*, *TNFRSF9*, *HAVCR2*, and *LAG3*) were used to score T-cell exhaustion [23]. 

Gene set enrichment analysis (GSEA) software (version 4.0.3) was used to identify pathways related to *SPAG9* [24,25]. The target set “C2.cp.kegg.v7.5.1.symbols.gmt” was downloaded from the Molecular Signatures database. The false discovery rate *p* value (FDR *p* value) and normalized enrichment score (NES) were used as screening criteria, and pathways with |NES| > 2 and FDR *p* values ≤ 0.05 were considered to be significantly enriched. Genes that contribute to the enrichment score (ES) of a particular pathway are called core enrichment genes, i.e., the leading-edge subset. According to the official description of GSEA, these genes can be interpreted as the core of a pathway and are, therefore, biologically important [25]. Based on the core enrichment genes of each pathway, we used the single-sample gene set-enrichment analysis (ssGSEA) algorithm to evaluate the pathway activity in samples.

### 2.6. Analysis with R Software

The R software (version 3.5.1) with the packages survival and survminer was used for the survival analysis. The Kaplan–Meier method was used to plot the survival curve, and log-rank was set as the statistical significance test. In Cox regression analysis, HRs were calculated based on the Cox PH Model. In the process of digitizing gender data, “female” was set as 0 and “male” was set as 1. Packages ggplot2 and ggpubr were used for the boxplots; Wilcoxon rank sum was set as the statistical significance test. The ratio of immune and stromal components in tumor samples was calculated by the ESTIMATE algorithm [26]. The heatmaps were plotted with the package Pheatmap. Correlation analysis was performed with the cor() function; Pearson’s correlation test served as the statistical significance test.

### 2.7. ScRNA-seq Data Processing

The ScRNA-seq data were initially processed by the package Seurat in R software. The percentage of mitochondrial genes was calculated by the PercentageFeatureSet() function. The correlations between sequencing depth and mitochondrial gene sequences and between sequencing depth and total intracellular sequences were calculated. Genes detected in <3 cells, cells with <200 total detected genes, cells with <50 sequencing numbers, and cells with ≥5% mitochondrial gene sequences were excluded. After filtering, LogNormalize() was used to normalize the data; FindVariableFeatures() was used to identify the top 1500 hypervariable genes. Principal component analysis (PCA) was performed, and under the condition of *p* value < 0.05, dimensions with significant separation were screened out [27]. The 20 principal components (PCs) were selected for secondary dimensionality reduction through the tSNE algorithm [28]. Marker genes in each cell population were identified with the criteria of |log2[fold change(FC)]| > 1 and *p* value < 0.05. Cell populations were annotated by the package SingleR and manually corrected with the CellMarker database (http://biocc.hrbmu.edu.cn/CellMarker/index.jsp/ accessed on 15 June 2022) based on the marker genes [29,30]. Pseudotime trajectories were constructed by the package Monocle2; annotated cell populations were positioned to specific locations [31].

### 2.8. Prognostic Model Construction and Validation

The TCGA cohort (515 patients) was set as the training cohort for constructing the prognostic model, and the ICGC cohort (91 patients) was set as the validation cohort for external validation. The RiskScore was constructed with lasso regression using the R package glmnet [32]. RiskScore = ExpGENE1 × β1 + ExpGENE2 × β2 + … + ExpGENEn × βn, in which “Exp” represents the expression level of the corresponding gene and “β” represents the regression coefficient calculated by multivariate Cox analysis [33]. Age, gender, and RiskScore were utilized to construct the nomogram by using the R package rms. Calibration plots were constructed to evaluate the predictive abilities of the nomograms; the number of resampling was 1000 [34]. The receiver operating characteristic (ROC) curves were plotted using the R package timeROC. Time-dependent C-indexes were calculated and plotted using the pec package.

### 2.9. Statistical Significance

A *p* value < 0.05 was considered statistically significant.

## 3. Results

### 3.1. SPAG9 Expression Suggests Poor Prognosis in Pan-Cancer Patients but Good Prognosis in ccRCC Patients

**Figure 1:**
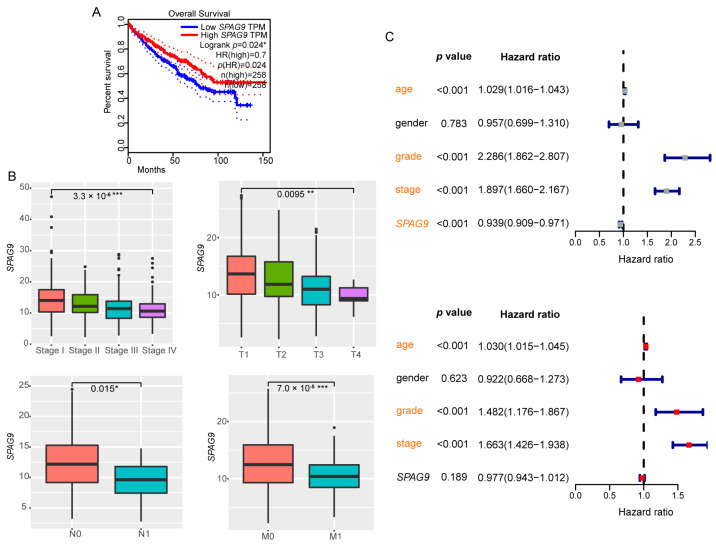
Survival analysis of *SPAG9* in ccRCC. (**A**) Kaplan–Meier survival analysis of 516 ccRCC patients was performed using GEPIA2 online tool. Median *SPAG9* TPM was chosen as the group cutoff. The 95% CI is shown by the dotted lines. (**B**) The *SPAG9*-expression differences between stages, T, N, and M in ccRCC patients. Wilcoxon rank-sum test served as the statistical significance test. (**C**) Univariate (**top**) and multivariate (**bottom**) Cox regression analyses of *SPAG9* expression and other clinicopathologic variables in ccRCC. Significant results are marked in orange. * *p* value < 0.05, ** *p* value < 0.01, *** *p* value < 0.001.

First, we explored the relationship between *SPAG9* expression and the clinical outcomes of the ccRCC patients. In ccRCC, the *SPAG9* expression was significantly associated with good prognosis (Figure 1A). Reduced *SPAG9* expression was significantly associated with clinical-stage progression (Stage I vs. Stage IV), primary tumor enlargement (T1 vs. T4), and tumor metastasis (N0 vs. N1 and M0 vs. M1) in the ccRCC patients (*p* value < 0.05, Figure 1B). The univariate Cox analysis showed that age (HR = 1.029, 95% CI = 1.016~1.043), pathological grade (HR = 2.286, 95% CI = 1.862~2.807), and clinical stage (HR = 1.897, 95% CI = 1.660~2.167) were risk factors for OS in ccRCC patients, and that *SPAG9* expression (HR = 0.939, 95% CI = 0.909~0.971) was a protective factor. The multivariate Cox analysis showed that age (HR = 1.030, 95% CI = 1.015~1.045), pathological grade (HR = 1.482, 95% CI = 1.176~1.867), and clinical stage (HR = 1.663, 95% CI = 1.426~1.938) were independent risk factors for OS in the ccRCC patients (*p* value < 0.05, Figure 1C).

**Figure 2:**
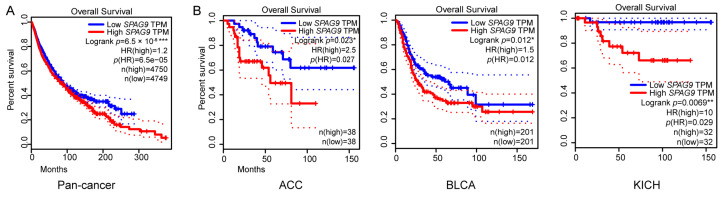
Survival analysis of *SPAG9* in pan-cancer. (**A**) Using GEPIA2 online tool, Kaplan–Meier survival analysis of 31 kinds of tumor patient, counting up to 9549, was performed. Median *SPAG9* TPM was chosen as the group cutoff. The 95% CI is shown by the dotted lines. (**B**) Using GEPIA2 online tool, Kaplan–Meier survival analysis was performed on all tumor types except for ccRCC. Only significant results are shown. Abbreviations: ACC, adrenocortical carcinoma; BLCA, bladder urothelial carcinoma; KICH, kidney chromophobe. * *p* value < 0.05, ** *p* value < 0.01, *** *p* value < 0.001.

In pan-cancer, *SPAG9* expression was significantly associated with poor prognosis (Figure 2A). In all the tumor types except for ccRCC, *SPAG9* was significantly correlated with OS in adrenocortical carcinoma (ACC), BLCA, and kidney chromophobe carcinoma (KICH), and it was a risk factor (Figure 2B).

### 3.2. SPAG9 Increases the Expression of Autophagy-Related Genes in 786-O Cells, but Not in HTB-9 Cells

Our study found that *SPAG9* expression predicted opposite clinical outcomes in the pan-cancer and ccRCC patients. To explore the underlying mechanism, we investigated the roles of *SPAG9* in ccRCC and bladder urothelial carcinoma (BLCA). The latter was chosen for comparison with ccRCC to represent the tumor types in which *SPAG9* expression suggests poor prognosis. The *SPAG9* expression was significantly positively correlated with OS in the ccRCC patients and significantly negatively correlated with OS in the BLCA patients. Previous studies showed that *SPAG9* promotes proliferation and migration in both ccRCC and BLCA cell lines [4,6,14], while *SPAG9* plays opposite prognostic roles in these two cancers. Therefore, we speculated that *SPAG9* participates in other physiological processes in ccRCC. 

We carefully reviewed previous studies on the role of *SPAG9* in different tumor-cell lines and summarized the results [4,5,6,7,8,9,10,11,12,13,14,15,16,17,18,19] (Table S5). We noted that in addition to promoting proliferation, migration, and EMT, *SPAG9* also promotes autophagy in several tumor-cell lines [18,19]. In ccRCC, increased autophagic flux was reported to be associated with better prognoses [35,36]; therefore, we speculated that *SPAG9* participates in autophagy in ccRCC. 

**Figure 3:**
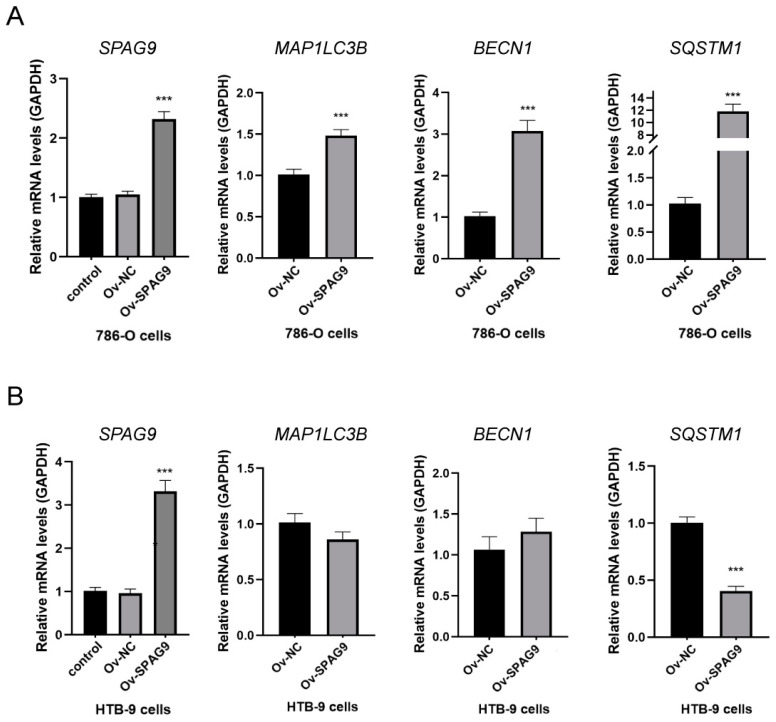
Effects of *SPAG9* overexpression on autophagy-related genes in (**A**) 786-O cells and (**B**) HTB-9 cells. Control: untreated cells; Ov-NC: cells transfected with control plasmids; Ov-*SPAG9*: cells transfected with *SPAG9* plasmids. *** *p* value < 0.001.

The molecules LC3B, Beclin1, and p62 are landmarks in the autophagic process [37], and the intensity of autophagy was assessed by examining the expression levels of these genes. We overexpressed *SPAG9* in the 786-O cells and HTB-9 cells and detected the expression levels of *MAP1LC3B* (LC3B), *BECN1* (Beclin1), and *SQSTM1* (p62) by RT–qPCR. The overexpression of *SPAG9* significantly increased the *MAP1LC3B*, *BECN1*, and *SQSTM1* mRNA levels in the 786-O cells (Ov-NC vs. Ov-*SPAG9*, *p* value < 0.05, Figure 3A), but not in the HTB-9 cells (Figure 3B).

### 3.3. SPAG9 Expression Was Significantly Correlated with a Weaker Inflammatory Response in ccRCC but Not in BLCA

**Figure 4:**
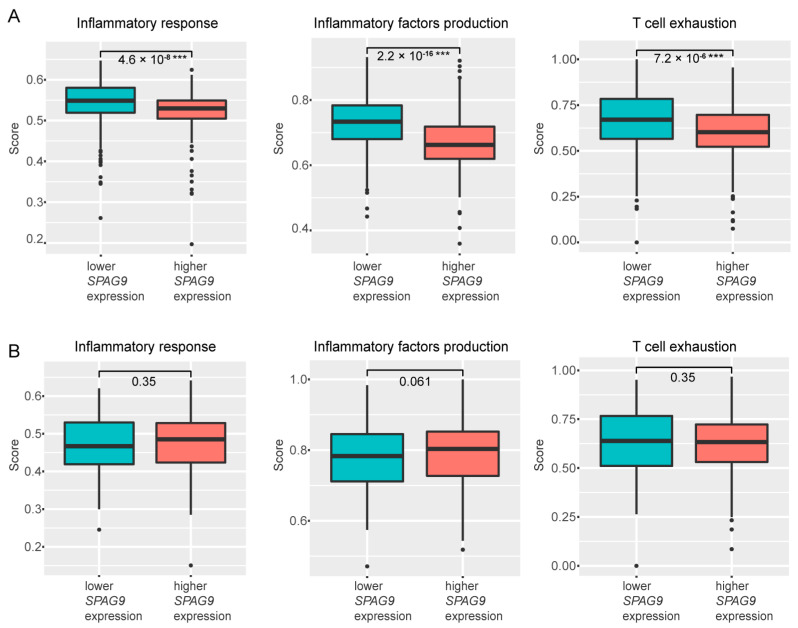
The scores of inflammatory response, inflammatory factor production, and T-cell exhaustion in (**A**) ccRCC tissues and (**B**) BLCA tissues with different levels of *SPAG9* expression. Median SPAG9 expression was chosen as the group cutoff. *** *p* value < 0.001.

Previous studies showed that autophagy activation inhibited tumor progression by suppressing the inflammatory response [38], so we investigated the relationship between *SPAG9* and the inflammatory response in ccRCC and BLCA. We used the ssGSEA method to evaluate the inflammatory response, inflammatory factor production, and T-cell-exhaustion levels in the ccRCC and BLCA samples. As shown in Figure 4, *SPAG9* expression was significantly correlated with a weaker inflammatory response, fewer inflammatory factors, and a lower degree of T-cell exhaustion in ccRCC (lower *SPAG9* expression vs. higher *SPAG9* expression, *p* value < 0.05, Figure 4A), but not in BLCA (Figure 4B).

### 3.4. The Correlation between SPAG9 Expression and ccRCC Prognosis Depends on the Expression of Key Genes

To further explore the role of *SPAG9* in ccRCC, we calculated the ratio of the immune to the stromal components in the ccRCC samples by using the ESTIMATE algorithm. The ccRCC samples were divided into three groups, based on the median immune and stromal scores: a stroma-rich group (samples with StromalScore > median StromalScore and ImmuneScore < median ImmuneScore), an immunocyte-rich group (samples with StromalScore < median StromalScore and ImmuneScore > median ImmuneScore), and a high-tumor-purity group (samples with StromalScore < median StromalScore and ImmuneScore < median ImmuneScore). A Kaplan–Meier survival analysis was conducted on the three groups, and the *SPAG9* expression was significantly associated with OS only in the stroma-rich group (Figure S1).

**Figure 5:**
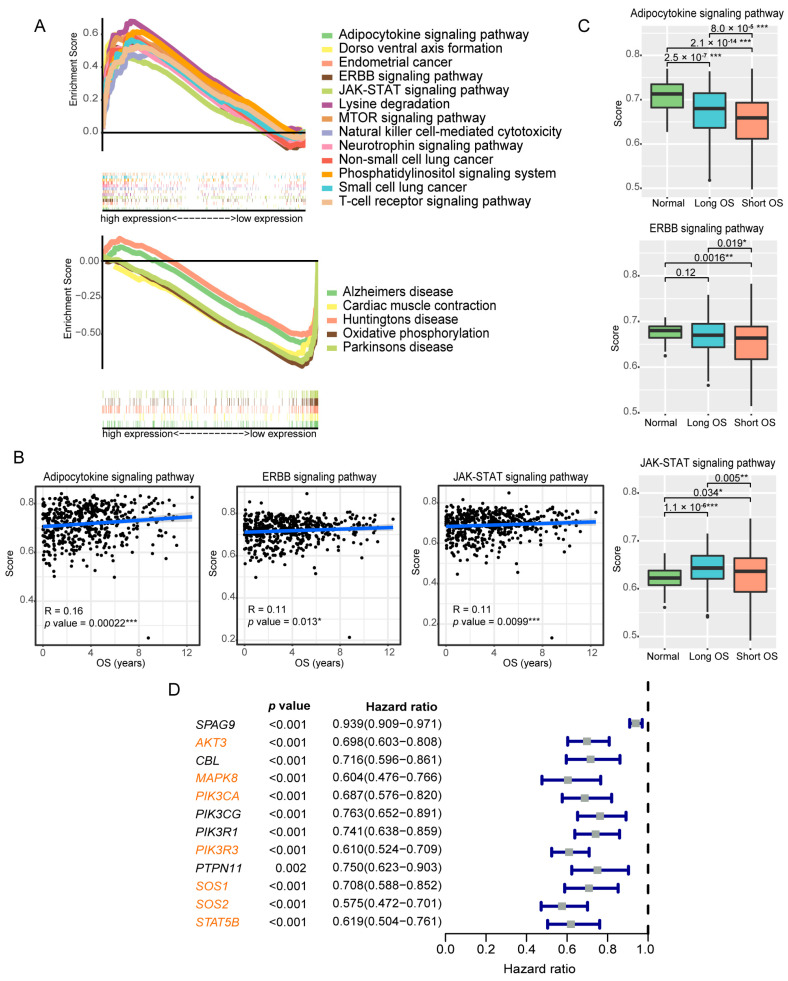
Analysis of the stroma-rich group and key-gene screening. (**A**) Tissues from the stroma-rich group were divided into two groups based on the median *SPAG9* expression (the high-*SPAG9*-expression group and the low-*SPAG9*-expression group), and GSEA analysis (in KEGG collection) was conducted, respectively. Each line represents one particular pathway with a unique color. At the bottom, genes up-regulated in a particular pathway are on the left of x-axis; by contrast, the down-regulated genes are on the right. Only pathways with |NES| > 2 and FDR *p* values ≤ 0.05 are shown. Top: the enriched pathways in the high-*SPAG9*-expression group; bottom: the enriched pathways in the low-*SPAG9*-expression group. (**B**) Scatter plots showing the correlation between the activity scores of the adipocytokine-signaling pathway (**left**), ERBB-signaling pathway (middle), and JAK-STAT-signaling pathway (**right**) and OS in ccRCC. (**C**) The activity scores of the adipocytokine-signaling pathway (**up**), ERBB-signaling pathway (**middle**), and JAK-STAT-signaling pathway (**down**) in tissues with different levels of malignancy. Normal: ccRCC paracancerous tissues; Long OS: ccRCC tissues from patients with longer OS (OS > median OS); Short OS: ccRCC tissues from patients with shorter OS (OS < median OS). (**D**) Univariate Cox analysis of the expression of *SPAG9* and candidate genes in ccRCC. The selected key genes are marked in orange. * *p* value < 0.05, ** *p* value < 0.01, *** *p* value < 0.001.

**Figure 6:**
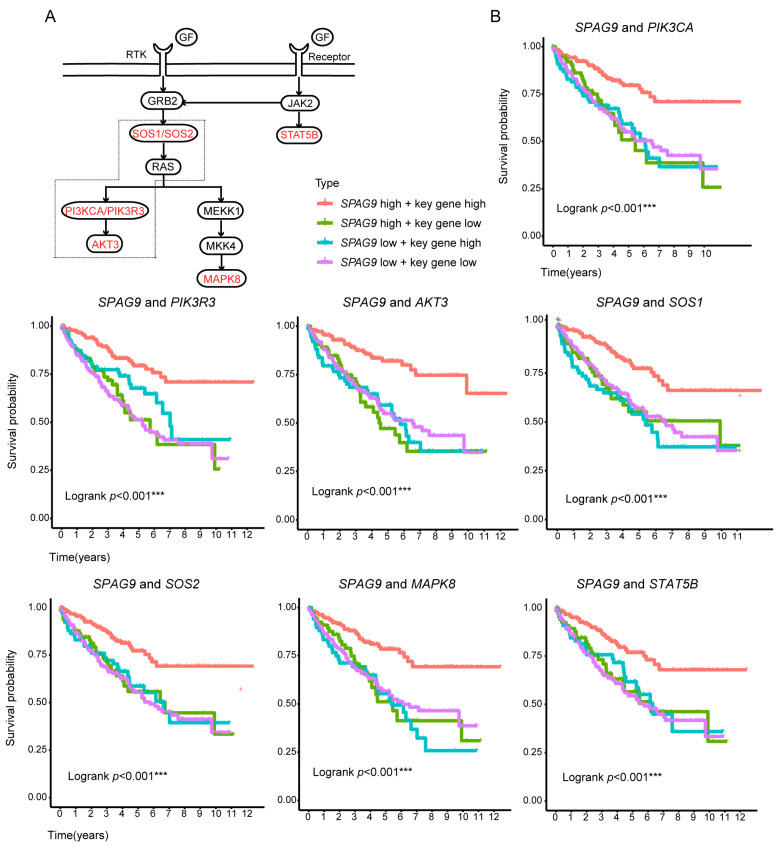
The correlation between *SPAG9* expression and ccRCC prognosis depends on the expression of key genes. (**A**) A signaling diagram collated from KEGG database. Key genes are marked in red. PI3K/AKT-pathway members are depicted by dotted lines. (**B**) Kaplan–Meier survival analysis was conducted on ccRCC patients with different gene-expression combinations (red: high key-gene expression and high *SPAG9* expression; blue: high key-gene expression and low *SPAG9* expression; green: low key-gene expression and high *SPAG9* expression; purple: low key-gene expression and low *SPAG9* expression). *** *p* value < 0.001.

Therefore, we performed the following analyses on the stroma-rich group. (1) The samples were divided into two groups based on the median *SPAG9* expression, and GSEA was conducted. As shown in Figure 5A, the genes in the high-*SPAG9*-expression group were significantly enriched in transmembrane signal transduction and phosphorylation regulatory pathways (adipocytokine-signaling pathway, ERBB-signaling pathway, JAK-STAT-signaling pathway, mTOR-signaling pathway, natural-killer-cell-mediated cytotoxicity, neurotrophin-signaling pathway, phosphatidylinositol-signaling system, and T-cell-receptor-signaling pathway), and the genes in the low-*SPAG9*-expression group were significantly enriched in mitochondrial dysfunction and abnormal calcium signaling pathways (Alzheimer’s disease, cardiac muscle contraction, Huntington’s disease, oxidative phosphorylation, and Parkinson’s disease). (2) Based on the core enrichment genes of each pathway, we used the ssGSEA algorithm to evaluate the pathway activity, and the correlation between the pathway activity and the OS was then calculated. Among the pathways listed above, the activities of the adipocytokine-signaling pathway, ERBB-signaling pathway, and JAK-STAT-signaling pathway were the most strongly correlated with the OS in the ccRCC patients, and these three pathways shared many common core enrichment genes (Figure 5B,C, Table S6). (3) From the common core enrichment genes of these three pathways, seven key genes (*AKT3*, *MAPK8*, *PIK3CA*, *PIK3R3*, *SOS1*, *SOS2*, and *STAT5B*) were screened out, and the expression of these genes had a high correlation with both the *SPAG9* expression and the OS in ccRCC (Figure 5D). The workflow for the screening of these key genes is shown in Table S7. In ccRCC, only the patients with high *SPAG9* expression/high key-gene expression had a better prognosis (Figure 6).

By analyzing the scRNA-seq data of the ccRCC patients, we found that the key genes were highly expressed in the tumor stromal cells (endothelial cells) (Figure S2), which was consistent with the previous finding that *SPAG9* expression was significantly associated with OS only in the stroma-rich group (Figure S1).

### 3.5. The Key Genes May Have a Synergistic Effect with SPAG9 in Terms of Promoting Autophagy

**Figure 7:**
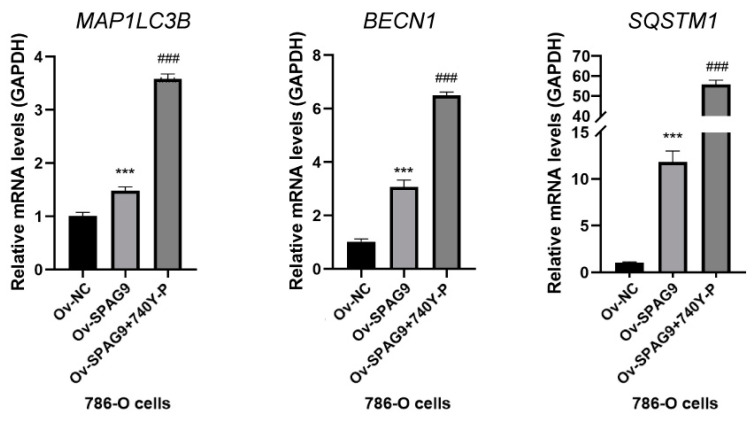
Effects of 740Y-P stimulation on autophagy-related genes in 786-O cells. *** *p* value < 0.001 vs. Ov-NC; ### *p* value < 0.001 vs. Ov-*SPAG9*. Ov-NC: cells transfected with control plasmids; Ov-*SPAG9*: cells transfected with *SPAG9* plasmids; Ov-*SPAG9* + 740Y-P: cells transfected with *SPAG9* plasmids and stimulated by 740Y-P.

Since most of the key genes belonged to the PI3K-AKT pathway (Figure 6A), to demonstrate the synergistic relationship between *SPAG9* and key genes, the PI3K agonist 740Y-P was used to stimulate the 786-O cells to mimic the effect of key-gene overexpression. The 740Y-P further increased the *MAP1LC3B*, *BECN1*, and *SQSTM1* mRNA levels by more than twofold compared with Ov-*SPAG9* cells (Ov-*SPAG9* + 740Y-P vs. Ov-*SPAG9*, *p* value < 0.05, Figure 7)

### 3.6. Construction and Validation of a SPAG9-Based ccRCC Prognostic Model

**Figure 8:**
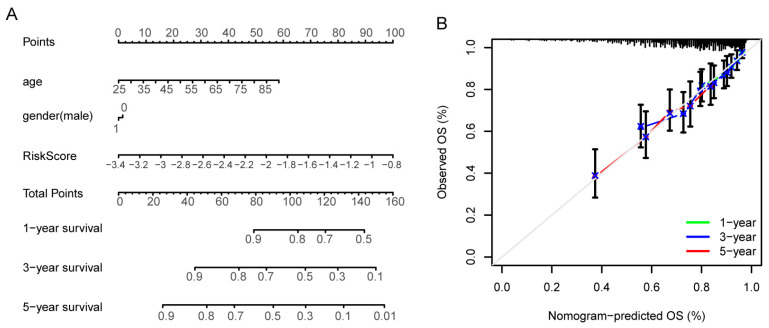
Construction of the *SPAG9*-based ccRCC prognostic model. (**A**) A nomogram to predict the prognoses of ccRCC patients, containing age, gender, and risk score. Total Points = (0.899 × age −22.468) + (−1.508 × gender + 1.508) + (38.462 × RiskScore + 130.769). (**B**) Calibration plots of the nomogram for predicting OS at 1-, 3-, and 5-year survival rates. T, actual survival proportion, is plotted on the y-axis with 95% confidence. Nomogram-predicted survival probability is plotted on the x-axis. The gray line is an ideal curve, with a slope of 1.

Based on the expression levels of *SPAG9*/key genes, we constructed a formula for the risk score by lasso regression: RiskScore (RS) = Exp*AKT3* × (−0.007) + Exp*PIK3R3* × (−0.337) + Exp*SOS1* × 0.196 + Exp*SOS2* × (−0.357) + Exp*STAT5B* × (−0.001). Next, we integrated age, gender, and RiskScore to construct a nomogram to predict the survival probability of the ccRCC patients (Figure 8A). The calibration curves showed good agreement between the predicted 1-, 3-, and 5-year survival rates and the respective actual survival rates (Figure 8B).

**Figure 9:**
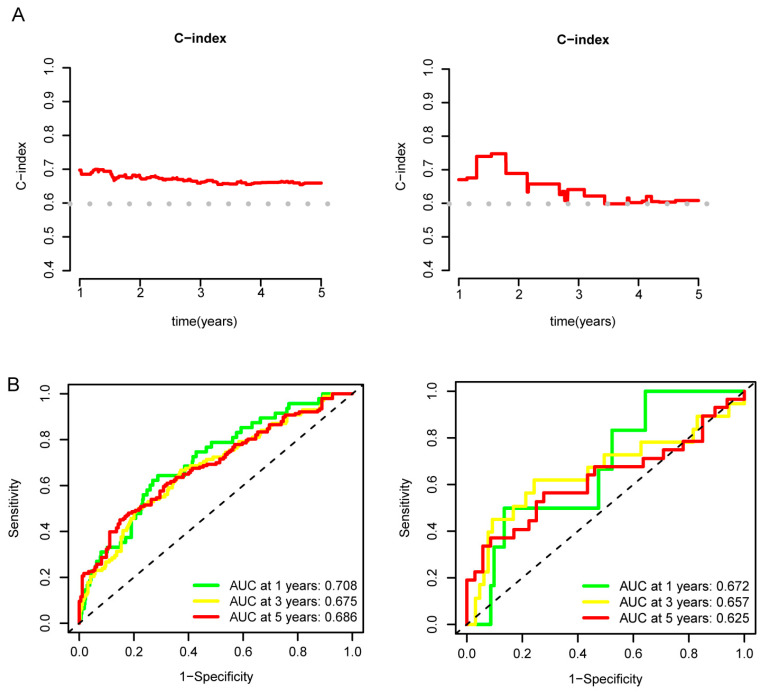
Validation of the *SPAG9*-based ccRCC prognostic model. (**A**) Time-dependent C-indexes of the nomogram in the training cohort (**left**) and validation cohort (**right**). The dotted lines were set at C-index = 0.6. (**B**) The ROC curves of the nomogram in the training cohort (**left**) and validation cohort (**right**). The dotted lines were reference lines, ROC curves under the reference line had no diagnostic value.

The predictive power of the nomogram was validated using C-indexes and ROC curves. The C-indexes of the nomogram were higher than 0.6 in both the training cohort and the validation cohort, and they were higher for the patients with shorter disease courses (Figure 9A). The ROC analysis indicated that the nomogram had some predictive value in both the training cohort and the validation cohort, especially in the patients with a 1-year disease course (in the training cohort: AUC at 1 years = 0.708, AUC at 3 years = 0.675, AUC at 5 years = 0.686; in the validation cohort: AUC at 1 years = 0.672, AUC at 3 years = 0.657, AUC at 5 years = 0.625, Figure 9B).

## 4. Discussion

It was reported that *SPAG9* promotes proliferation and migration in many tumor-cell lines, including the ccRCC cell lines Caki-1 and NII-AKS413 [4]. However, we found that *SPAG9* was associated with good prognosis in ccRCC patients, which contrasted with its widely recognized cancer-promoting role and was not found in other tumors. Thus, we speculated that *SPAG9* participates in other physiological processes in ccRCC.

We summarized the roles of *SPAG9* in tumor-cell lines and noted that in addition to promoting proliferation, migration, and EMT [4,5,6,7,8,9,10,11,12,13,14,15,16,17], *SPAG9* also promotes autophagy in several tumor-cell lines [18,19]. Autophagy is a cellular degradation pathway for macromolecular substances. Cells initiate autophagy after they are stimulated by external stress, and misfolded proteins and damaged organelles are engulfed by autophagic vesicles and lysosomes [37]. Autophagy has a dual effect in tumors: in the early stage, autophagy inhibits tumorigenesis by inhibiting the destruction of the genome by the reactive oxygen species produced by damaged mitochondria. In the late stage, autophagy helps tumor cells survive hypoxia and a lack of nutrients [38,39,40]. 

In ccRCC, Radovanovic et al. found that an increase in autophagic flux was associated with a lower tumor stage, reduced metastasis, and an improved 5-year survival rate [36]. Xu also found that the autophagy-promoting gene MAP1S was associated with better prognoses in ccRCC patients [35]. In ccRCC, studies reported that the activation of autophagy effectively inhibits tumor progression [41,42]. Moreover, prognostic models based on autophagy have also been widely developed and proven to have good predictive value [43,44,45,46,47]. Thus, autophagy may play an important role in ccRCC and, overall, it has a protective effect. 

Autophagy inhibits inflammatory responses by inhibiting oxidative stress and lysosomal rupture [38]. Inflammation plays an important role in tumor progression: inflammatory responses stimulate tumor-associated macrophages to secrete cytokines, such as TGF-β and TNF-α, and promote tumor-cell metastasis; a persistent inflammatory response also leads to T-cell exhaustion and further tumor progression [22]. Clear-cell renal cell carcinoma is a tumor type with a high degree of immune infiltration [1]. The activation of autophagy in ccRCC may reduce the pro-inflammatory stress from tumor cells to the surrounding immune cells, inhibit tumor progression, and prolong the survival times of patients.

Therefore, we hypothesized that *SPAG9* suppresses tumor progression by promoting autophagy in ccRCC. We found that the overexpression of *SPAG9* significantly increased the mRNA levels of autophagy-associated genes in the 786-O cells but not in the HTB-9 cells. Moreover, the *SPAG9* expression was significantly correlated with weaker inflammatory responses, fewer inflammatory factors, and a lower degree of T-cell exhaustion in ccRCC, but not in BLCA. Based on this information, we established a possible mechanism through which *SPAG9* affects the prognosis of patients with ccRCC and BLCA: in ccRCC, *SPAG9* promotes tumor growth but also promotes autophagy and inhibits the inflammatory response, and it has a protective effect on the whole; in BLCA, SPAG9 promotes tumor growth and leads to poor prognoses.

Through further analyses, we found that the correlation between *SPAG9* expression and ccRCC prognosis was dependent on the tumor stroma. By analyzing stroma-rich samples, we identified seven key genes (*AKT3*, *MAPK8*, *PIK3CA*, *PIK3R3*, *SOS1*, *SOS2*, and *STAT5B*) in this study. In ccRCC, *SPAG9* expression was significantly associated with OS only when key genes were highly expressed. The scRNA-seq data further proved that the key genes were highly expressed in the tumor stroma of ccRCC. Since most of the key genes were PI3K-AKT-pathway members, we used the PI3K agonist 740Y-P to stimulate the 786-O cells to mimic the effect of key-gene overexpression. Furthermore, the addition of 740Y-P significantly increased the expression of the autophagy-related genes by more than twofold compared with the Ov-*SPAG9* cells. In conclusion, we speculated that in the tumor stroma, some genes cooperate with *SPAG9* to promote autophagy, and that these may be key genes, which can be represented by the key genes.

Finally, we constructed a nomogram based on the *SPAG9*/key genes and other clinical features to predict survival in the ccRCC patients. Combined with external data validation, the nomogram proved to have some predictive value. The estimation of the long-term prognosis of ccRCC patients can help physicians develop individualized treatment plans [48]. With this new nomogram, we look forward to providing more helpful guidance for clinical work.

## 5. Conclusions

In recent years, research on the role of *SPAG9* in tumors has mainly focused on its role in promoting tumor progression [49], but our study found that *SPAG9* expression was significantly associated with good prognoses in ccRCC, a result that has not been found in other cancer types and contradicts the widely recognized cancer-promoting role of *SPAG9*. Combined with a bioinformatics analysis and an experimental validation, we speculated that *SPAG9* suppresses tumor progression by promoting autophagy and inhibiting inflammatory responses in ccRCC. We further found that some genes might cooperate with *SPAG9* to promote autophagy, and that these were highly expressed in the tumor stroma and may be key genes. Finally we constructed a nomogram based on *SPAG9*/key genes and other clinical features, which was proven to have some predictive value.

## Data Availability

The data that support the findings of this study are openly available on the TCGA database and the GEO database.

## Supplementary Materials

The following supporting information can be downloaded at: https://www.mdpi.com/article/10.3390/genes14040944/s1. Figure S1: *SPAG9* expression is significantly associated with OS only in the stroma-rich group. Figure S2: The key genes are highly expressed in ccRCC stromal cells. Table S1: Baseline clinical characteristics of ccRCC patients from the TCGA database. Table S2: Baseline clinical characteristics of ccRCC patients from the ICGC database. Table S3: Primers designed for this study. Table S4: The qPCR data after analysis with 2^−ΔΔCt^ method. Table S5: The role of *SPAG9* in different tumor-cell lines; Table S6: The correlation between the activity scores of pathways and OS in ccRCC. Table S7: Workflow for screening of key genes.
