# Supplementary material for: SPAG9 Expression Predicts Good Prognosis in Patients with Clear-Cell Renal Cell Carcinoma: A Bioinformatics Analysis with Experimental Validation
Source: Genes (Basel). 2023 Apr 20;14(4):944. doi: 10.3390/genes14040944 (PMC10138117; doi:10.3390/genes14040944)
Supplement: Supplementary file 1 [file genes-14-00944-s001.zip › Legends for supplementary figures.pdf]

Legends for supplementary figures:

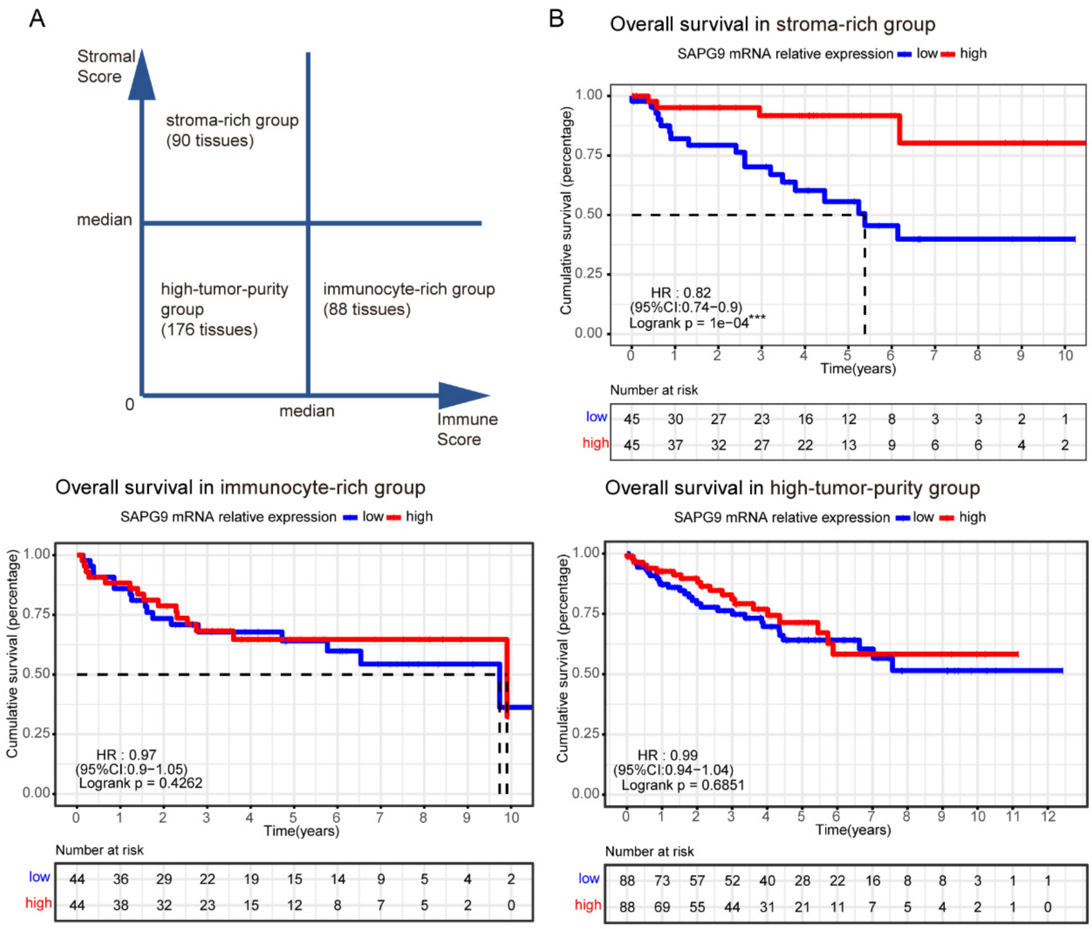

Figure S1. SPAG9 expression is significantly associated with OS only in the stroma-rich group. (**A**) Based on the median ImmuneScore and StromalScore, 538 samples were divided into 3 groups: a stroma-rich group (samples with StromalScore > median StromalScore and ImmuneScore < median ImmuneScore), an immunocyte-rich group (samples with StromalScore < median StromalScore and ImmuneScore > median ImmuneScore) and a high-tumor-purity group (samples with StromalScore < median StromalScore and ImmuneScore < median ImmuneScore). (**B**) Kaplan−Meier survival analysis was conducted in stroma-rich group (top), immunocyte-rich group (left bottom) and high-tumor-purity group (right bottom). Median SPAG9 expression was chosen as the group cutoff.

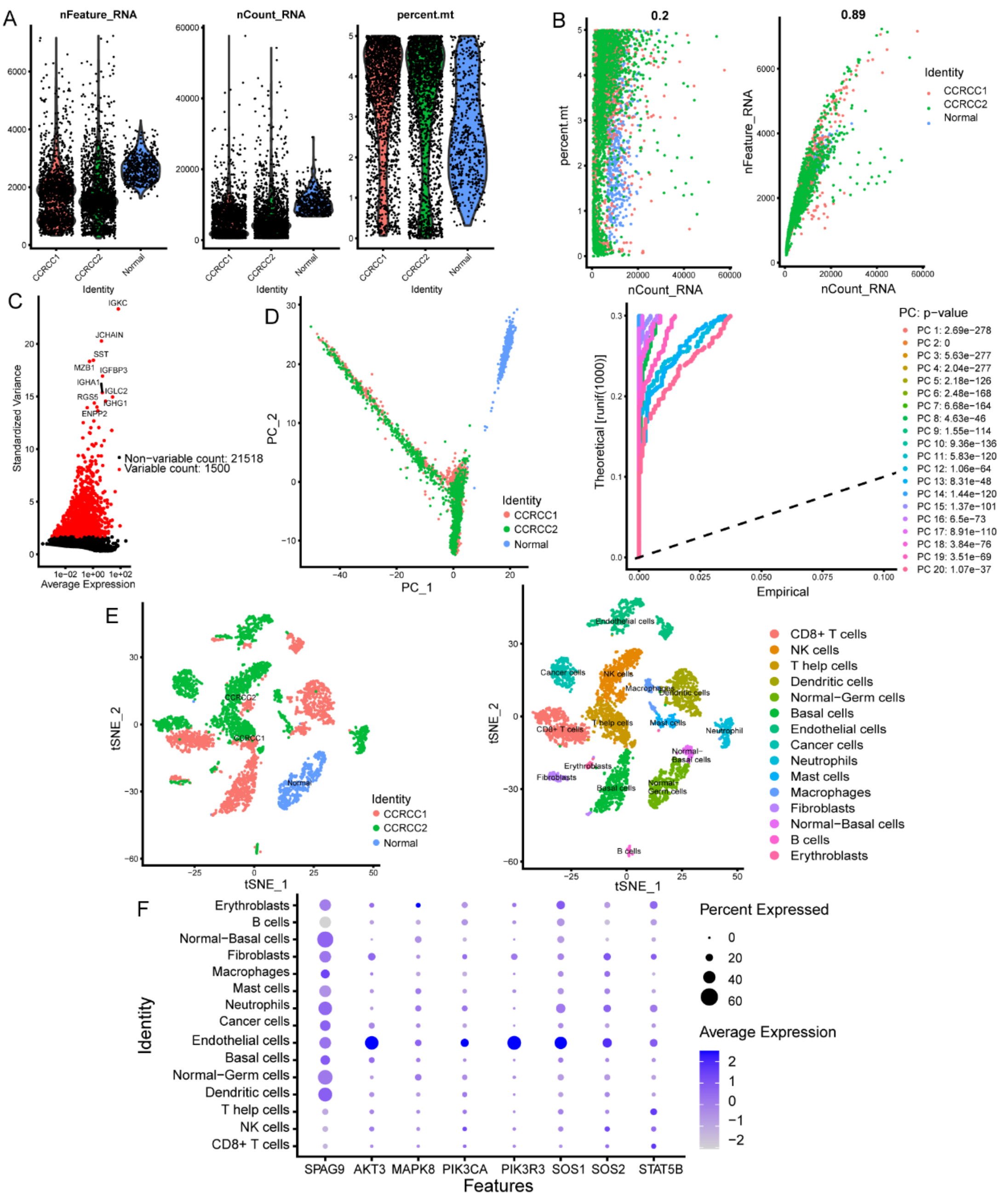

Figure S2. The key genes are highly expressed in ccRCC stromal cells. (**A**) The quality control of GSE152938 took gene numbers, cell numbers and mitochondrial gene numbers into consideration. 4464 cells were included in the subsequent analysis. (**B**) Sequencing depth was not correlated with mitochondrial gene sequences but significantly correlated with total intracellular sequences, with a Pearson’s correlation coefficient of 0.89. (**C**) The variance diagram showed 23018 corresponding genes, of which 1500 hypervariable genes were marked red. Names of the top 10 most hypervariable genes were shown. (**D**) PCA showed significant segregation of ccRCC cells and normal kidney cells (left). 20 PCs with significant differences were selected for the subsequent analysis (right). (**E**) After t-SNE dimensionality reduction, the cell populations were annotated by singleR and CellMarker. (**F**) The relative expression levels of SPAG9 and key genes in different cell types.
