# Supplementary material for: SPAG9 Expression Predicts Good Prognosis in Patients with Clear-Cell Renal Cell Carcinoma: A Bioinformatics Analysis with Experimental Validation
Source: Genes (Basel). 2023 Apr 20;14(4):944. doi: 10.3390/genes14040944 (PMC10138117; doi:10.3390/genes14040944)
Supplement: Supplementary file 1 [file genes-14-00944-s001.zip › Table S1-S7.pdf]

Table S1. Baseline clinical characteristics of ccRCC patients from the TCGA database. Data was presented as number (percentage) or mean ± SD.

Total patients (n=515)	
Age (years)	60 ± 12
Gender (male)	336 (65%)
Grade	
1	13 (3%)
2	224 (43%)
3	205 (40%)
4	73 (14%)
Stage	
I	257 (50%)
II	54 (10%)
III	122 (24%)
IV	82 (16%)
fustat (alive)	346 (67%)
futime (years)	4 ± 3

Table S2. Baseline clinical characteristics of ccRCC patients from the ICGC database. Data was presented as number (percentage) or mean ± SD.

Total patients (n=91)	
Age (years)	60 ± 10
Gender (male)	52 (57%)
fustat (alive)	61 (67%)
futime (years)	4 ± 2

Table S3. Primers designed for this study.

Genes	Forward sequence	Reverse sequence
GAPDH	TGACATCAAGAAGGTGGTGAAGCAG	GTGTCGCTGTTGAAGTCAGAGGAG
SPAG9	GCTGTGGTGTGCTGTTGGAGTC	TCGCTGTTTACTGCCTTCTGTATCC
MAP1LC3B (LC3B)	GTCAGCGTCTCCACACCAATCTC	ACAATTTCATCCCGAACGTCTCCTG
BECN1 (Beclin1)	AGGCATGGAGGGGTCTAAGG	AATGGCTCCTGTGAGTTCCTG
SQSTM1 (p62)	GATAGCCTTGGAGTCGGTGG	TCAGCCTCTGTAGATGGGTCC

Table S4. The qPCR data after analysis with 2-ΔΔCt method.

Figure 3A (786-O cells):

Overexpression of *SPAG9*:

Control	Ov-NC	Ov-SPAG9
1.098092814	1.218410264	1.898684242
1.04608494	1.017479692	2.505328877
1.098092814	1.113421618	2.470837274
0.781869643	1.07549439	2.006943497
1.031683179	0.837987135	2.611719574
0.982820599	1.060687741	2.453769955

MAP1LC3B expression in 786-O cells:

Ov-NC	Ov-SPAG9
0.958156574	1.482809572
1.093030254	1.775632885
1.273089047	1.589235949
1.019833287	1.383510251
0.828362031	1.327151742
0.887816443	1.336382813

BECN1 expression in 786-O cells:

Ov-NC	Ov-SPAG9
0.967052867	3.098707688
1.110852038	3.84149178
1.445598252	3.634272673
0.896059642	3.141964035
0.727826591	2.516932745
0.987372704	2.191117219

SQSTM1 expression in 786-O cells:

Ov-NC	Ov-SPAG9
0.796456055	9.010871634
0.90229223	14.94578394
0.841868418	13.46989469
1.015131529	14.74002092
1.538651675	9.073547203
1.058239861	9.724787103

Figure 3B (HTB-9 cells):

Overexpression of *SPAG9*:

Control	Ov-NC	Ov-SPAG9
0.820741609	0.7888329	3.041957506
0.885767519	0.7237016	2.918040688
0.942784536	1.110741	4.184339759
1.244011653	1.0948551	3.986161051
1.28788163	0.7795067	2.648177821
0.910669834	1.282356	3.1058755

MAP1LC3B expression in HTB-9 cells:

Ov-NC	Ov-SPAG9
1.406068292	1.110852038
0.967052867	0.68380964
0.927659117	0.764011716
0.927659117	0.748288582
0.960372945	1.001155913
0.889870106	0.859557863

BECN1 expression in HTB-9 cells:

Ov-NC	Ov-SPAG9
1.620755722	1.84889932
0.502315837	1.032875715
1.122462048	1.228303149
1.107008782	0.676736762
1.245449622	1.522736872
0.793700526	1.391524844

SQSTM1 expression in HTB-9 cells:

Ov-NC	Ov-SPAG9
0.96761162	0.571371111
0.87812608	0.364123561
1.081100018	0.289673577
1.088619663	0.376964351
	0.464097554
	0.364123561

Figure 7 (786-O cells):

MAP1LC3B expression in 786-O cells:

Ov-NC	Ov-SPAG9	Ov-SPAG9+740Y-P
0.958156574	1.482809572	3.526735386
1.093030254	1.775632885	3.651105441
1.273089047	1.589235949	3.859284319
1.019833287	1.383510251	3.383070715
0.828362031	1.327151742	3.313448126
0.887816443	1.336382813	3.779861397

BECN1 expression in 786-O cells:

Ov-NC	Ov-SPAG9	Ov-SPAG9+740Y-P
0.967052867	3.098707688	6.505530315
1.110852038	3.84149178	6.240521808
1.445598252	3.634272673	6.550779856
0.896059642	3.141964035	6.28392807
0.727826591	2.516932745	6.924292488
0.987372704	2.191117219	

SQSTM1 expression in 786-O cells:

Ov-NC	Ov-SPAG9	Ov-SPAG9+740Y-P
0.796456055	9.010871634	57.74670269
0.90229223	14.94578394	64.96841783
0.841868418	13.46989469	56.55829274
1.015131529	14.74002092	49.92417446
1.538651675	9.073547203	52.40623885
1.058239861	9.724787103	53.13780268

Table S5. The role of *SPAG9* in different tumor cell lines

The role of *SPAG9*	Cancer type	Cell lines used in the study	References
Promoting proliferation	Hepatocellular carcinoma	HepG2 cells [16];<br>QGY cells [15]	[15-16]
	Ovarian cancer	A10, SKOV-3 and Caov3 cells [13];<br>SKOV3-ip, COV362, OVCAR4 and ES-2 cells [9]	[9, 13]
	Gastric cancer	HGC-27 and SGC-7901 cells [8];	[8]
	Osteosarcoma	U2OS cells [11]	[11]
	Prostate cancer	PC-3, DU145 and C4-2 cells [17];<br>HGC-27 cells and SGC-7901 cells [7]	[7, 17]
	Bladder urothelial carcinoma (BLCA)	*UM-UC-3 cells [6]*	[6]
	Clear cell renal carcinoma (ccRCC)	*Caki-1 and NII-AKS413 cells [4]*	[4]
	Colorectal Cancer	COLO 205 and HCT 116 cells [5]	[5]
	Triple-negative breast cancer	MDA-MB-231 cells [10]	[10]
Promoting migration	Hepatocellular carcinoma	QGY cells [15]	[15]
	Ovarian cancer	SKOV3-ip, COV362, OVCAR4 and ES-2 cells [9]	[9]
	Gastric cancer	HGC-27 and SGC-7901 cells [8]	[8]
	Osteosarcoma	U2OS cells [11-12]	[11-12]
	Prostate cancer	PC-3, DU145 and C4-2 cells [17];<br>HGC-27 cells and SGC-7901 cells [7]	[7, 17]
	Bladder urothelial carcinoma (BLCA)	*UM-UC-3 cells [6];*<br>*T24 and 5637 cells [14]*	[6]<br>[14]
	Clear cell renal carcinoma (ccRCC)	*Caki-1 and NII-AKS413 cells [4]*	[4]
	Colorectal Cancer	COLO 205 and HCT 116 cells [5]	[5]
	Triple-negative breast cancer	MDA-MB-231 cells [10]	[10]
Promoting EMT	Ovarian cancer	A10, SKOV-3 and Caov3 cells [13]	[13]
	Triple-negative breast cancer	MDA-MB-231 cells [10]	[10]
	Bladder urothelial carcinoma (BLCA)	*T24 and 5637 cells [14]*	[14]

Promoting Angiogenesis	Osteosarcoma.	U2OS cells [12]	[12]
Inhibiting apoptosis	Hepatocellular carcinoma	HepG2 cells [16]	[16]
	Ovarian cancer	A10, SKOV-3 and Caov3 cells [13]	[13]
	Triple-negative breast cancer	MDA-MB-231 cells [10]	[10]
**Promotion autophagy**	Cervical cancer	HeLa cells [18-19]	[18-19]
	Colorectal Cancer	HCT-116 cells [18]	[18]
	Hepatocellular carcinoma	HepG2 cells [18]	[18]
	Glioma	U87 cells [19]	[19]
	Fibrosarcoma	HT1080 cells [19]	[19]

Studies with overexpression/knockdown of *SPAG9* in tumor cell lines were included. Studies in ccRCC and BLCA were marked in italics. Abbreviation: EMT, epithelial-mesenchymal transition.

Table S6. The correlation between the activity scores of pathways and OS in ccRCC

Pathways	Correlation analysis with OS in ccRCC	
	R (Correlation coefficient)	p value
Adipocytokine signaling pathway	0.16	<0.001***
Dorso ventral axis formation	0.093	0.032*
Endometrial cancer	0.1	0.019*
ERBB signaling pathway	0.11	0.013*
JAK-STAT signaling pathway	0.11	0.0099**
Lysine degradation	0.083	0.055
MTOR signaling pathway	0.096	0.027*
Natural killer cell-mediated cytotoxicity	0.1	0.015*
Neurotrophin signaling pathway	0.098	0.024*
Non-small cell lung cancer	0.077	0.078

Phosphatidylinositol signaling system	0.052	0.23
Small cell lung cancer	0.083	0.057
T-cell receptor signaling pathway	0.09	0.039*
Alzheimers disease	0.0072	0.87
Cardiac muscle contraction	0.0018	0.97
Huntingtons disease	0.0017	0.97
Oxidative phosphorylation	0.0018	0.97
Parkinsons disease	-0.00077	0.99

The activity scores of the adipocytokine signaling pathway, ERBB signaling pathway and JAK-STAT signaling pathway were most strongly correlated with OS in ccRCC patients; these 3 pathways were marked in italics.

Table S7. Workflow of screening key genes

Candidate genes	Correlation analysis with *SPAG9* expression p value < 0.05		Kaplan–Meier survival analysis p value (log-rank) < 0.05	Univariate Cox analysis HR < 0.71 p value (HR) < 0.05	
	R (Correlation coefficient)	p value	p value (log-rank)	HR	p value (HR)
AKT3	0.62	<0.001***	<0.001***	0.698	<0.001***
CBL	0.64	<0.001***	0.0041**	0.716	<0.001***
CBLB	0.43	<0.001***	0.87		
JAK2	0.51	<0.001***	0.06		
MAPK8	0.77	<0.001***	<0.001***	0.604	<0.001***
MTOR	0.47	<0.001***	0.23		
PIK3CA	0.68	<0.001***	<0.001***	0.687	<0.001***

PIK3CD	0.24	<0.001***	0.27		
PIK3CG	0.34	<0.001***	<0.001***	0.763	<0.001***
PIK3R1	0.44	<0.001***	<0.001***	0.741	<0.001***
PIK3R3	0.46	<0.001***	<0.001***	0.610	<0.001***
PTPN11	0.63	<0.001***	0.0011**	0.750	0.002**
SOS1	0.74	<0.001***	<0.001***	0.708	<0.001***
SOS2	0.72	<0.001***	<0.001***	0.575	<0.001***
STAT3	0.62	<0.001***	0.12		
STAT5B	0.71	<0.001***	<0.001***	0.619	<0.001***

The candidate genes listed in the first column were the common core enriched genes of the adipocytokine signaling pathway, ERBB signaling pathway and JAK-STAT signaling pathway, and the screening steps were listed in the first row with their conditions. Screening was carried out step by step. If one gene did not meet the conditions of a certain step, it would be excluded from the process. The first 2 steps were performed with GEPIA2. All analyses were carried in ccRCC samples. 7 key genes were ultimately screened out and were marked in bold.
